# Photodynamic Therapy with Aminolevulinic Acid Enhances the Cellular Activity of Cells Cultured on Porcine Acellular Dermal Matrix Membranes Used in Periodontology

**DOI:** 10.3390/gels9070584

**Published:** 2023-07-20

**Authors:** Morena Petrini, Emira D’Amico, Tania Vanessa Pierfelice, Gitana Maria Aceto, Maryia Karaban, Pietro Felice, Adriano Piattelli, Antonio Barone, Giovanna Iezzi

**Affiliations:** 1 Department of Medical, Oral and Biotechnological Sciences, University G. d’Annunzio of Chieti-Pescara, 66100 Chieti, Italy; emira.damico@unich.it (E.D.); tania.pierfelice@unich.it (T.V.P.); gitana.aceto@unich.it (G.M.A.); gio.iezzi@unich.it (G.I.); 2 Department of Biomedical and Neuromotor Sciences, University of Bologna, 40126 Bologna, Italy; maryia.karaban@studio.unibo.it (M.K.); pietro.felice@unibo.it (P.F.); 3 School of Dentistry, Saint Camillus International University of Health and Medical Sciences, 00131 Rome, Italy; apiattelli51@gmail.com; 4 Department of Surgical, Medical, Molecular Pathologies and of the Critical Needs, School of Dentistry, University of Pisa, 56126 Pisa, Italy; antonio.barone@unipi.it; 5 Complex Unit of Stomatology and Oral Surgery, University Hospital of Pisa, 56126 Pisa, Italy

**Keywords:** photodynamic therapy, aminolevulinic acid, red light, dermal matrix membrane, gingival fibroblasts, oral osteoblasts

## Abstract

This study aims to test a photodynamic protocol based on a gel containing aminolevulinic acid followed by red-LED (ALAD-PDT) irradiation on human gingival fibroblasts (hGFs) and osteoblasts (hOBs) cultured on a porcine acellular dermal matrix membrane (PADMM). In the previous literature, ALAD-PDT showed solid antibacterial activity and proliferative induction on HGFs cultured on plates and HOBs cultured on a cortical lamina. PADMMs are used in dentistry and periodontology to treat gingival recessions and to increase the tissue thickness in the case of a thin biotype without the risks or postoperative discomfort associated with connective tissue grafts. However, one of the possible complications in this type of surgery is represented by bacterial invasion and membrane exposition during the healing period. We hypothesized that the addition of ALAD-PDT to PADMMs could enhance more rapid healing and decrease the risks connected with bacterial invasion. In periodontal surgery, PADMMs are inserted after a full-thickness flap elevation between the bone and the flap. Consequently, all procedures were performed in parallel on hOBs and hGFs obtained by dental patients. The group control (CTRL) was represented by the unexposed cells cultured on the membranes, group LED (PDT) were the cells subjected to 7 min of red LED irradiation, and ALAD-PDT were the cells subjected to 45 min of ALAD incubation and then to 7 min of red LED irradiation. After treatments, all groups were analyzed for MTT assay and subjected to histological examination at 3 and 7 days and to the SEM observations at 3, 7, and 14 days. Different bone mineralization assays were performed to quantify the effects of ALAD-PDT on hOBs: ALP activity, ALP gene expression, osteocalcin, and alizarin red. The effects of ALAD-PDT on hGFs were evaluated by quantifying collagen 1, fibronectin, and MMP-8. Results showed that ALAD-PDT promoted cellular induction, forming a dense cellular network on hOBs and hGFs, and the assays performed showed statistically significantly higher values for ALAD-PDT with respect to LED alone and CTRLs. In conclusion, ALAD-PDT could represent a promising aid for enhancing the healing of gingival tissues after PADMM applications.

## 1. Introduction

Aminolevulinic acid (5-ALA) is a precursor of protoporphyrin IX, a photosensitive substance that, after activation with red light at specific wavelengths, enhances the production of free radicals and singlet oxygen molecules [1]. It is currently applied in dermatology for medical and cosmetic purposes, including treating precancerous lesions [2]. The 5-ALA-mediated photodynamic therapy (PDT) is a non-invasive emerging method in dentistry as a diagnostic and therapeutic tool [3]. It has been demonstrated that ROS production is the critical factor in the effectiveness of PDT, and it depends on light dose and photosensitizer concentrations [4,5]. However, a recent study has demonstrated better tolerability of 5-ALA at a concentration of less than 20%, representing the indicated concentration PDT guidelines for managing oral leucoplakia [6,7]. In addition, the hydrophilic nature of 5-ALA limits its ability to cross the cellular membranes and penetrate the skin [8]. However, a novel gel for oral cavity applications has been recently formulated with a mixture of poloxamers to convey its active ingredient better. This thermosensitive gel containing 5% of 5-delta aminolevulinic acid (ALAD), associated with red-LED irradiation (ALAD-PDT), has shown antimicrobial activity against Gram-negative and positive bacteria against *C. albicans* and oral biofilm [9,10,11]. Clinical case reports have recently been published that show the application of ALAD-PDT during root canal disinfection in endodontic treatments and in periodontology [12,13]. In particular, for the treatment of periodontal pockets and peri-implantitis sites, the addition of this protocol respects the traditional gold standard scaling root planning (SRP), and was associated with a reduction in the total bacterial load. Despite the antibacterial and anticancer activity of 5-ALA-PDT, a pro-regenerative nature of 5-ALA-mediated PDT is recently emerging in the literature. Several studies reported the capacity of photodynamic therapy to accelerate skin wound healing by promoting epithelial stem cell functions [14,15,16,17]. However, little is known about the potential healing effects of 5-ALA-PDT in periodontal-related tissues. Our previous study shows a pro-proliferative activity of the gel ALAD in osteoblasts (hOBs) and fibroblasts (hGFs) on cell culture on plates and a cortical membrane for bone regeneration. Results showed increased hOBs proliferation, ALP activity, and bone mineralization proportional to ALAD concentration [18,19]. Several efforts have been made to find a biomaterial to be used in tissue and bone regeneration [20,21]. During periodontal and bone regeneration procedures, the membrane and the biomaterial interact with the host’s cells, and their chemical and surface features play a fundamental role in accelerating the healing of the tissues and long-term success. It has been shown that the topographical characteristics of Porcine acellular dermal matrix membranes (PADMs) can influence cellular proliferation and behavior [22]. PADMs are composed of a three-dimensional acellular network of collagen types I and III and elastin of heterologous origin. PADMs permit the treatment of gingival recessions or to increase the tissue thickness in the case of a thin biotype without the risks or postoperative discomfort associated with connective tissue grafts (CTGs). Therefore, based on our previous in vitro studies, we hypothesized that applying ALAD-PDT protocol to PADMMs could enhance the healing process. At the same time, the risks connected with bacterial invasion could be decreased. In periodontal surgery, PADMMs are inserted after a full-thickness flap elevation between the bone and the flap. Consequently, this study evaluated the response of hOBs and hGFs, extracted from dental patients, cultured on the membranes, and subjected to ALAD-PDT. The effects of ALAD-PDT were investigated in terms of adhesion, growth, mineralization activity, and gene expression.

## 2. Results and Discussion

### 2.1. Cell Attachment on the PADMM after ALAD-PDT

**Figure 1:**
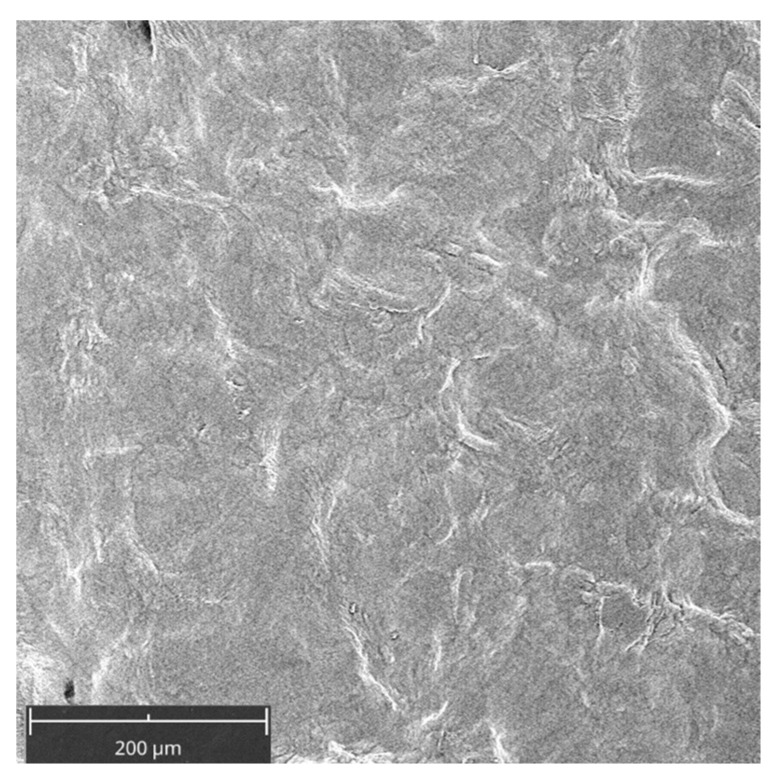
Scanning electron microscopy (SEM) images of the membrane (PADMM) without cells. Magnification 390×.

**Figure 2:**
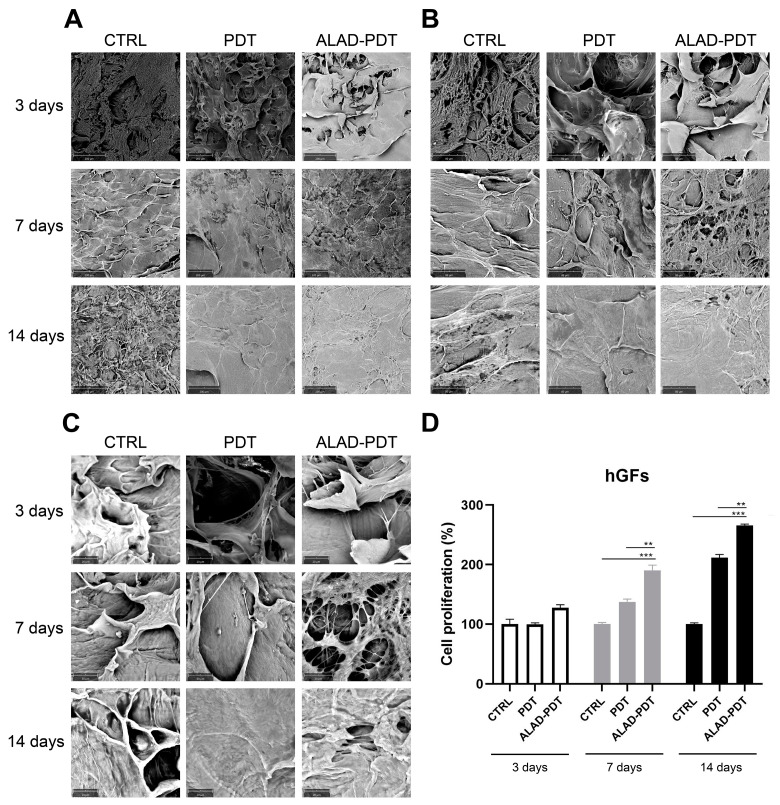
Scanning electron microscopy (SEM) images of hGFs cultured on the PADMM and exposed to ALAD-PDT at 3, 7, and 14 days. (**A**) Magnification 390×; (**B**) Magnification 1000×; (**C**) Magnification 3000×; (**D**) Cell proliferation of hGFs cultured on the PADMM at 3, 7, and 14 days. (** *p* < 0.001; *** *p* < 0.0001).

**Figure 3:**
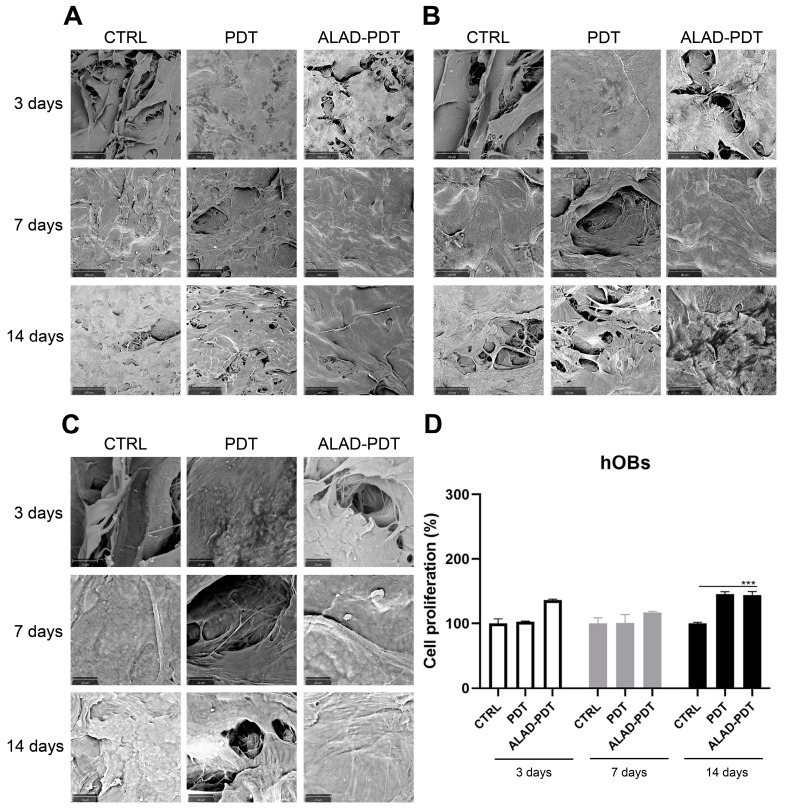
Scanning electron microscopy (SEM) images of hOBs cultured on the PADMM and exposed to ALAD-PDT at 3, 7, and 14 days. (**A**) (Magnification = 390×; (**B**) Magnification, 1000×; (**C**) Magnification and 3000×; (**D**). Cell proliferation of hOBs cultured on the PADMM at 3, 7, and 14 days. (*** *p* < 0.0001).

Figure 1 shows the membrane without cells. Its surface appeared smoothed. Cell adhesion was evaluated by SEM at 3, 7, and 14 days (Figure 2 and Figure 3). At 3 days, HGFs colonized the surface of the matrix, especially after the ALAD-PDT protocol, as observed in the different magnifications (Figure 2). At 1000×, the presence of elongated and spindle-shaped with cytoplasmic extensions and lamellipodia were recognizable. Confluence was reached after 7 days; at 14 days, the cells covered the matrix entirely. At 14 days, the morphology of cells was not notable because of the high density of cells.

Regarding hOBs, ALAD-PDT promoted the attachment of cells to the membrane at 3 days, as observed at each magnification (Figure 3). After 7 days of culture, hOBs colonized the matrix surface. At 3000×, cellular extensions among osteoblasts were observed at 3 and 7 days. In contrast, at 14 days, it was impossible to recognize the cells’ morphology because of their full confluence reached. The increased adhesion of cells to the PADMM membrane after ALAD-PDT may be due to the formulation of ALAD gel based on the mixture of poloxamers. Indeed, the topical application of 5-ALA is limited by its hydrophilic nature, preventing it from entering easily into the cells. The poloxamers-rich compound permits the thermosensitive gel ALAD to improve this limiting characteristic and makes it ideal for the application of oral mucosa. In addition, the formulation is temperature-dependent, allowing the transient state of ALAD from liquid to gel at a temperature above 28 °C; thus, ALAD acts like a glue that increases the attachment of cells, and it also counteracts the continuous secretion of saliva that could obstacle the adhesion of cells.

### 2.2. Gingival Fibroblasts and Oral Osteoblast Proliferation

The proliferation rate of gingival fibroblasts and oral osteoblasts cultured on the PADMMs was evaluated by MTT at 3, 7, and 14 days (Figure 2 and Figure 3). As observed in Figure 2D, the growth of hGFs is time dependent. LED irradiation in the PDT group exerted a pro-proliferative activity compared to the control group after 7 and 14 days of culture. This is in line with a recent study that demonstrated that 655 and 808 nm diode lasers speed up the proliferation of dermal fibroblasts [23]. Recently, Rossi et al. documented an opposite effect, both inhibitory and stimulatory, in different doses, by treating dermal fibroblasts with a blue LED light at 420 nm [24]. In the present study, although LED was able to enhance cell proliferation, the maximum stimulatory effect was given by ALAD-PDT at each time. However, the results were statistically significant only at 7 and 14 days compared to CTRL and PDT groups. Jang et al. reported that PDT-induced intracellular ROS in dermal fibroblasts leads to increased proliferation via ERK pathway activation [25]. In hOBs, ALAD-PDT also increased cell growth, but not in a time-dependent manner (Figure 3D), and it was statistically significant only after 14 days. In the literature, a contradictory effect of 5-ALA-PDT on osteoblasts was reported. Kushibiki et al. demonstrated that 5-ALA, combined with low-dose light, can promote osteoblast differentiation via the activation of AP-1 [26]. On the contrary, Egli et al. showed an inhibitory effect of 5-ALA-PDT on fibroblasts and osteoblasts viability [27].

### 2.3. Cell Interaction with the PADMM after ALAD-PDT

**Figure 4:**
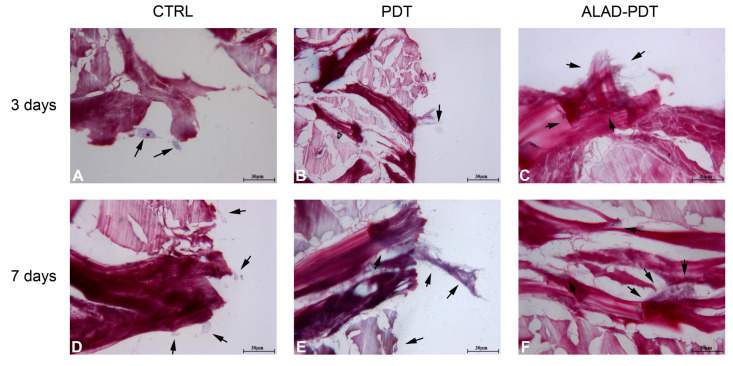
hGFs interaction with PADMM. hGFs grew on the edges of the membrane at 3 days (**A**–**C**). At 7 days, they colonize the inside of the membrane (**D**–**F**). Magnification: 400×. The arrows pointed to the cells.

**Figure 5:**
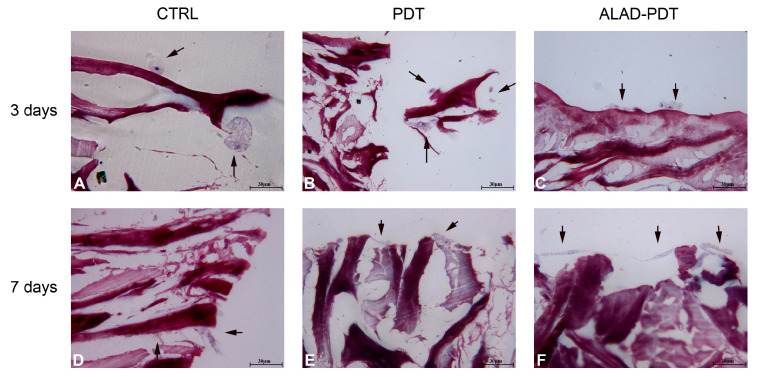
Interaction between hOBs and PADMM. At 3 days, osteoblast showed a round shape (**A**–**C**); at 7 days, they appeared more elongated (**D**–**F**). Magnification: 400×. The arrows pointed to the cells.

The interaction of specialized cells to a membrane is an essential characteristic of its physiological functions, and in this study, it was evaluated by histology at 400× at 3 and 7 days (Figure 4 and 5). At 3 days, after ALAD-PDT protocol, hGFs grew on the edges of the membrane by establishing connections among cells (Figure 4C). After 7 days, PDT also promoted the interaction of hGFs (Figure 4E), and after ALAD-PDT cells colonized the inside of the membrane (Figure 4F). At 3 days, hOBs appeared roundish in shape, and after 7 days their shape became elongated (Figure 5). A recent publication reported an inhibitory effect of toluidine blue-mediated PDT, used at a concentration of 50% on the migratory activity of gingival fibroblasts [28]. In contrast, the thermosensitive gel used in the present study and which contains 5% of 5-ALA as the pro-drug able to induce the accumulation of PpIX inside the cells, seemed to enhance the interaction of the cells and favor their migratory activity.

### 2.4. ALP Activity

**Figure 6:**
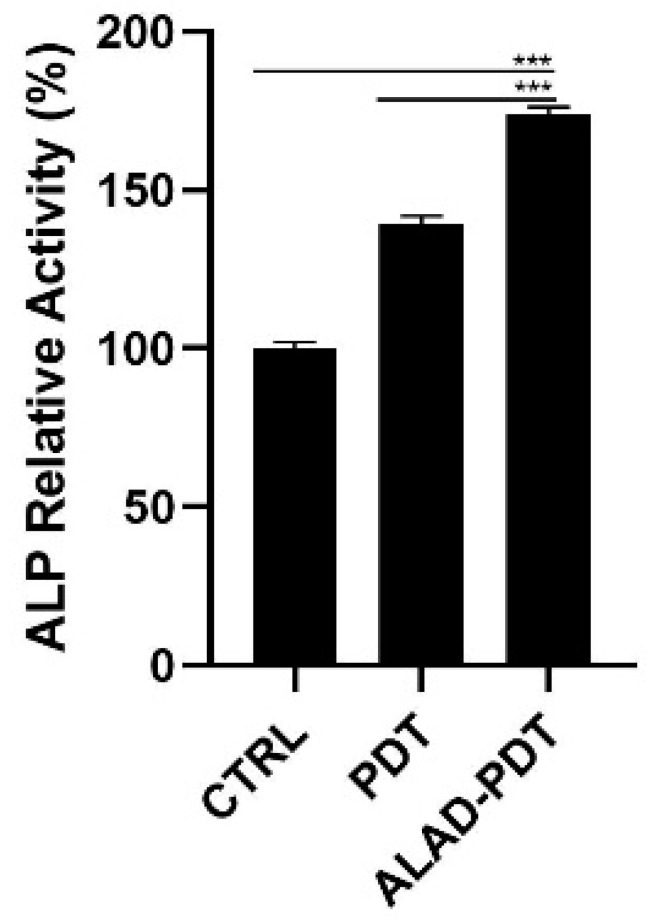
ALP activity of hOBs cultured for 7 days and exposed to ALAD-PDT protocol. Increased levels were observed after the treatment (*** *p* < 0.0001).

The levels of Alkaline Phosphatase (ALP), as the main osteoblastic marker, were evaluated at 7 days in hOBs (Figure 6). ALP relative activity increased after Led irradiation in the PDT group, although the ALAD-PDT induced a significative increment of ALP compared to both CTRL group (*p* < 0.0001) and to PDT group (*p* < 0.0001). Yang et al. reported a similar result by treating gingival fibroblasts with 5 μM methylene blue PDT. However, they obtained a higher ALP activity in the laser treatment group [29]. Whereas in our study, only the synergy between ALAD gel and light-LED significantly promoted the activity of ALP in osteoblasts.

### 2.5. Mineralization

**Figure 7:**
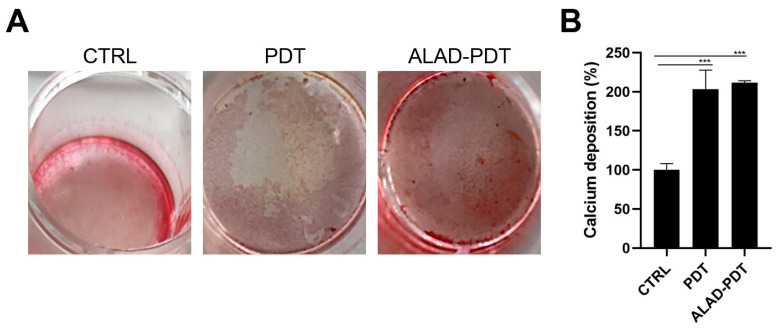
Mineralization was evaluated at 14 days in hOBs seeded on the membrane and exposed to ALAD-PDT. The qualitative analysis was performed by ARS (**A**), while the quantization was carried out by CPC (**B**). ALAD-PDT promoted the highest calcium deposits compared to CTRL. (*** *p* < 0.0001).

To determine the presence of calcific deposition in osteoblast cultures, calcium deposits were evaluated qualitatively by Alizarin Red staining (ARS) and quantitatively by CPC after 14 days of culture (Figure 7). Brighter red mineralized nodules were observed after ALAD-PDT protocol compared to CTRL (Figure 7A), indicating more mineralization activity in osteoblasts treated with ALAD-PDT. The PDT group also showed a similar intensity of red to the ALAD-PDT group. Quantization with CPC confirmed the qualitative results. Therefore, the percentage of calcium deposition was statistically higher in PDT and ALAD-PDT groups than in the CTRL group (*p* < 0.0001). However, there was no difference in calcium deposits between PDT and ALAD-PDT (Figure 7B). Yang et al. conversely observed more mineralized nodule formation in laser-treated cells [29]. In addition, in our previous study, an increase in the mineralization and calcium deposits was observed when osteoblasts were cultured on a cortical, rigid, and collagenated bone lamina and subjected to ALAD-PDT [30].

### 2.6. Gene Expression of Gingival Fibroblasts and Oral Osteoblast Cultured on the PADMM and Exposed to Photodynamic Therapy

**Figure 8:**
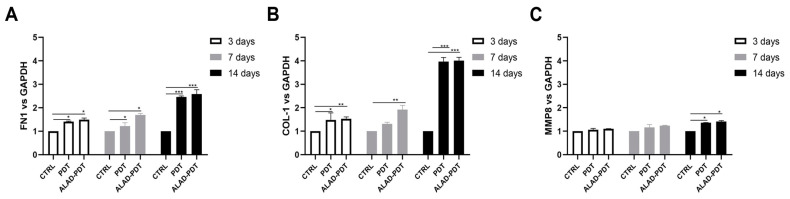
Real-time PCR of fibroblasts (HGFs) cultured on the matrix and treated with ALAD-PDT for Fibronectin 1 (FN1) (**A**), Collagen 1 (COL-1) (**B**), Metalloprotease 8 (MMP8) (**C**) at 3, 7, and 14 days post-seeding. ALAD-PDT induced increased expression of FN1, COL-1, and MMP8 at 14 days (* *p* < 0.05; ** *p* < 0.001; *** *p* < 0.0001).

**Figure 9:**
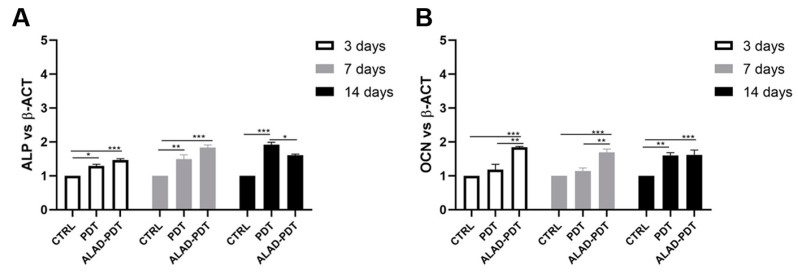
Real-time PCR of osteoblasts (hOBs) seeded on the PADMM and treated with ALAD-PDT for genes encoding Alkaline Phosphatase (ALP) (**A**) and Osteocalcin (OCN) (**B**) at 3, 7, and 14 days post-seeding. ALP and OCN were more expressed in the ALAD-PDT group than in CTRL. (* *p* < 0.05; ** *p* < 0.001; *** *p* < 0.0001).

The gene expression of gingival fibroblasts and oral osteoblasts cultured on the matrix and exposed to ALAD-PDT protocol was evaluated at 3, 7, and 14 days. Fibronectin 1 (FN1), Collagen 1 (COL-1), and metalloproteinase 8 (MMP8) expression were evaluated for hGFs (Figure 8), while ALP and osteocalcin (OCN) for hOBs (Figure 9). Fibronectin and Collagen I are well known to have a tight relationship both related to connective tissue regulation. The comparative analysis showed that the expression of FN1 and COL-1 was statistically higher in PDT and ALAD-PDT groups than in the CTRL group at every time point (Figure 8A,B). Although, the highest values for both FN1 and COL-1 have been observed at 14 days. Any statistical difference was observed between PDT and ALAD-PDT. MMP8 is a member of the metalloproteinase family, specifically involved in both the degradation of matrix and the wound-healing-processes [31]. MMP8 was not modulated at 3 and 7 days with respect to CTRL group (Figure 8C). ALAD-PDT and PDT slightly increased MMP8 expression only at 14 days (*p* < 0.05). Karrer et al. found that 5-ALA-mediated PDT induced in fibroblast the expression of metalloproteinases MMP1, MMP2, and MMP3 but not Collagen I mRNA expression [32]. In contrast, Yang et al. reported an enhancement of fibroblast-related genes FN1 and COL1 after applying 5 μM methylene blue-PDT on cells. Although, in the same study, an inhibitory effect on gene expression of fibroblasts treated with higher concentrations than 5 μM was reported [29]. Our results concerning FN1, COL1, and MMP8 expression could indicate a stimulatory effect of ALAD-PDT for the renewal of the extracellular matrix (ECM).

The expression of bone tissue-specific genes ALP and OCN, which are tightly regulated at different stages of osteoblasts, was evaluated at 3, 7, and 14 days (Figure 9). Although ALP gene expression was statistically augmented after ALAD-PDT protocol compared to CTRL at every time points, the expression was not modulated during time with the highest value at 7 days (Figure 9A). Interestingly, PDT group showed the maximum expression at 14 days. Osteocalcin expression is usually correlated to the ALP expression, and in this study, OCN showed a similar trend to ALP. OCN-relative mRNA was up-regulated in the ALAD-PDT group compared to CTRL and PDT, with the highest value at an early stage of osteoblasts. Although, at 14 days, OCN expression showed similar levels in PDT and ALAD-PDT groups (Figure 9B).

Together, these findings indicated that the ALAD-PDT protocol may be applied during guided tissue regeneration (GTR) and guided bone regeneration (GBR) to improve the performance of PADMM in the periodontal tissue’s augmentation. The thermosensitive ALAD gel, formulated to improve the topical application of 5-ALA on the oral mucosa, where the saliva represents an additional limit to the retention of 5-ALA, enhanced the adhesion and the interaction of periodontal-related cells cultured on the membrane.

The results of this in vitro study could represent the bases for an in vivo study in which ALAD-PDT could be applied as an adjuvant during GBR and GTR.

## 3. Conclusions

In conclusion, ALAD-PDT applied on the gingival fibroblasts and oral osteoblasts cultured on a porcine dermal matrix membrane promoted the proliferation, mineralization, and expression of functional genes such as FN1 and COL1 in hGFs, and ALP and OCN in hOBs. 

## 4. Materials and Methods

### 4.1. Study Design

The effects of the photodynamic protocol (ALAD-PDT) on the human gingival fibroblasts (hGFs) and human oral osteoblast (hOBs) cultured on the PADMM (Cellis Dental, La Rochelle, France) were performed using MTT assay, SEM and gene expression at 3, 7 and 14 days, histology at 3 and 7 days, Alkaline Phosphatase levels (ALP) at 7 days and Alizarin Red Staining (ARS) at 14 days. The following experimental conditions were used:

i. PDT: cells cultured on the membrane and exposed to 630 nm LED for 7 min;
ii. ALAD-PDT: cells cultured on the membrane and treated with a gel containing 5% of 5-aminolevulinic acid (ALAD) for 45 min and irradiated with red light (LED) for 7 min;
iii. And CTRL: cells cultured on the membrane PADM.

The PADMM, used in the in vitro tests, was cut into squares of 5 mm × 5 mm under sterilized conditions, and they were hydrated with NaCl 0.9% three times before the culture of cells.

Photodynamic therapy is based on the use of a gel containing 5-aminolevulinic acid at the concentration of 5% (ALAD) (AlphaStrumenti, Melzo (MI), Italy) and a 630 nm LED (PDT) (AlphaStrumenti, Melzo (MI), Italy). ALAD gel is a thermosetting product, protected by a patent (PCT/IB2018/060368, 12.19.2018), that remains liquid at temperatures below 28 °C, becoming gel at higher temperatures. Further, 630 nm LED has an intensity of 380 mW/cm^2^ with a light dose of 23 J/cm^2^.

### 4.2. Cell Culture

**Figure 10:**
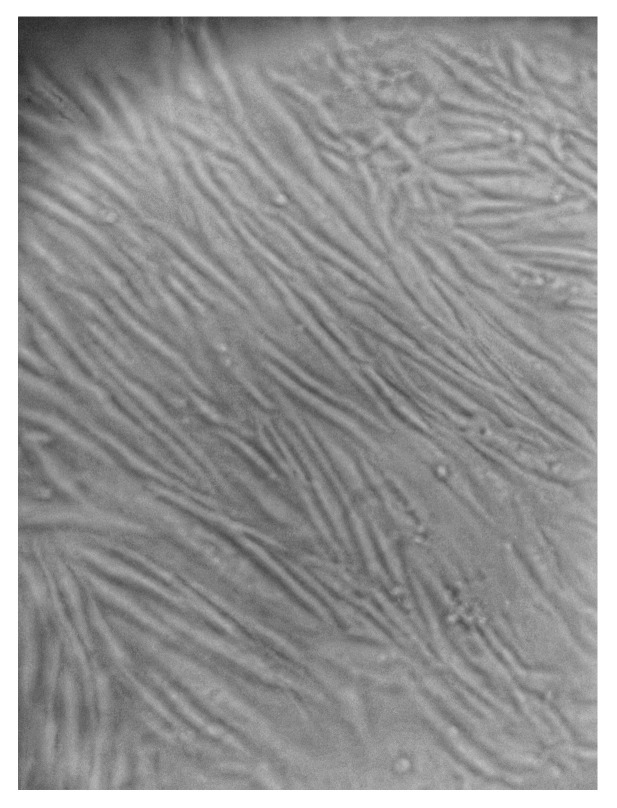
Human oral osteoblasts (hOBs) at optical microscopy at 5th passage. Magnification: 10×.

Primary human gingival fibroblasts (hGFs) were purchased from ATCC (Manassas, VA, USA), and human oral osteoblasts (hOBs) were extracted from bone fragments of patients treated at Dental Clinic of University Gabriele d’Annunzio (Ethical Committee reference numbers: BONEISTO N. 22 of 10.07.2021) according to the protocol described by Pierfelice TV and co-workers [33]. In particular, during the procedures of dental implant insertion, during the implant site preparation, a trephine bur was used to sample a bone fragment. Briefly, bone fragments were subjected to three enzymatic digestions at 37 °C for 20, 40, and 60 min using collagenase type 1A (Sigma-Aldrich, St. Louis, MO, USA) and trypsin-EDTA 0.25% (Corning, New York, NY, USA). After each digestion, this solution was centrifuged at 1200 rpm for 10 min, and the pellet obtained was transferred into a T25 culture flask with low-glucose (1 g/L) DMEM supplemented with 10% FBS (SIAL, Rome, Italy), 1% antibiotics (100 µg/mL^−1^ streptomycin and 100 IU/mL^−1^ penicillin), and 1% L-glutamine (Corning) at 5% CO_2_ and 37 °C. The medium was changed every 4–5 days. hGFs and hOBs were cultured in DMEM low glucose (Corning) supplemented with 10% fetal bovine serum (FBS) (SIAL), 1% penicillin, and streptomycin (Corning) at 37 °C and 5% CO_2_. Both cell lines, hOBs and hGFs, were used from the 3rd and 5th passages. Figure 10 shows hOBs at the optical microscope.

### 4.3. Cell Proliferation Assay

MTT evaluated the growth of cells seeded on the matrix. A total of 10^4^ cells/membrane were seeded on the matrix, exposed to ALAD-PDT, and cultured for 3, 7, and 14 days. The choice of cell density was based on previous studies and considering the area of the specimen [34,35]. Then, the MTT assay (Sigma Aldrich, St. Louis, MO, USA) was used according to the manufacturer’s instructions. At the end of each incubation period, a solution of 0.5 mg/mL MTT (Sigma Aldrich, St. Louis, MO, USA) was added to each well, and then the cells were incubated for 4 h at 37 °C and 5% CO_2_. A solubilization solution was added to each well to dissolve the insoluble formazan. Then, the plate was read at 570 and 630 nm by a microplate reader (Synergy H1 Hybrid BioTek Instruments, Winooski, VT, USA) to determine the absorbance (A). Then, a subtraction A = A570 − A630 was performed. The results were expressed as percentages and calculated with respect to control (CTRL).

### 4.4. Cell Attachment

The adhesion of cells was observed using scanning electron microscope (SEM) images. The cells at 10^4^ cells/membrane density were seeded on the matrix, treated with ALAD-PDT, and cultured for 3, 7, and 14 days. Loosely adherent cells were removed from the experiment wells by washing twice with 0.1 M PBS (pH 7.4). Thereafter, cells were fixed with 2.5% glutaraldehyde for 1 h and dehydrated using sequential concentrations of ethanol (40, 50, 75, 95, 100%). Before the observation, they were sputtered with gold and observed at 390×, 1000×, and 3000× using SEM (Philips XL20; Philips Inc., Eindhoven, the Netherlands) at 15 kV.

### 4.5. Histological Analysis

The interaction of cells with the membrane was evaluated by histological analysis. A total of 10^4^ cells/membranes were seeded on the top of the matrix. Cells were exposed to ALAD-PDT protocol, and the culture was carried out for 7 days. Each specimen was fixed with 10% buffered formalin and dehydrated in an ascending alcohol series. They were then polymerized in a glycol methacrylate resin (Technovit 7200 VLC; Kulzer, Wehrheim, Germany). The sections, about 30 µm in width, were stained with fuchsin and toluidine blue. The images were taken by an optical microscope (Leica, Wetzlar, Germany) at 400×.

### 4.6. ALP Assay

ALP assay was performed to evaluate ALP levels in hOBs using a colorimetric kit AB83369 (Abcam Inc., Cambridge, UK) based on the cleavage of *p*-nitrophenyl phosphate (pNPP). hOBs at a density of 5 × 10^4^ were seeded on top of the matrix, treated with ALAD-PDT, and cultured for 7 days. Thereafter, the assay was performed according to the manufacturer’s instructions. After 7 days, the cells were washed three times with PBS and resuspended in assay buffer. The cell suspension was then homogenized by a Tissue Rupture device (QIAGEN, Hilden, Germany) and centrifuged at 10,000× *g* for 15 min. The relative ALP activity of the supernatant was measured using pNPP, as the substrate, for 1 h. After incubation, the reaction was stopped and the relative ALP activity was quantified as an absorbance value at 405 nm. 

### 4.7. Mineralization

The deposition of calcium nodules was evaluated by ARS (Sigma Aldrich, St. Louis, MO, USA) and quantized with 10% Cetylpyridinium Chloride (CPC) solution (Sigma-Aldrich). Then, 5 × 10^4^ hOBs/membrane were seeded on the membrane, exposed to ALAD-PDT protocol, and cultured for 14 days. The cells were fixed with 2.5% glutaraldehyde and then stained with ARS solution for 1h. After 1 h, deionized water was used to remove the excess dye, and the presence of mineral nodules stained by red color was observed. Then, to quantize calcium deposits, 1 mL of 10% CPC solution was added, and after 1 h the absorbance was read at 540 nm in a microplate reader (Synergy H1 Hybrid BioTek Instruments).

### 4.8. Gene Expression

The gene expression of different markers of osteoblastic cells (ALP and OCN) and markers of fibroblast activity (COL1, FN1, and MMP8) were evaluated by RT-PCR. Total RNA was isolated using the Trifast reagent (EuroClone, Pero (MI), Italy), and RNA was quantified on a Nanophotometer NP80 spectrophotometer (Implen NanoPhotometer, Westlake Village, CA, USA) for analysis of RNA integrity, purity, and concentration. Then, the GoTaq^®^2 Step RT-qPCR Kit (Promega, Madison, WI, USA) was used to obtain complementary DNA (cDNA), and SYBR Green (GoTaq^®^ 2 Step RT-qPCR Kit, Promega) was used to perform RT-qPCR according to manufacturer’s instructions. Gene expression was determined using Quant Studio 7 Pro Real-Time PCR System (ThermoFisher, Waltham, MA, USA). The results were normalized to Glyceraldehyde-3-Phosphate Dehydrogenase (GAPDH for hGFs and to β-actin (β-ACT) for hOBs using the 2^−ΔΔct^ method. Primer sequences are reported in Table 1.

### 4.9. Statistical Analysis

The data are reported as means ± standard deviation (SD). Statistical analyses were performed using the GraphPad Prism8 (GraphPad Software San Diego, CA, USA), and ANOVA and post hoc Tukey tests were adopted. A *p*-value < 0.05 was considered significant. All experiments were performed in biological triplicates and repeated three times.

## Figures and Tables

**Table 1:** Primer sequences used in RT-qPCR.

Gene	Forward Primer (5′-3′)	Reverse Primer (5′-3′)
OCN	TCAGCCAACTCGTCACAGTC	GGCGCTACCTGTATCAATGG
ALP	AATGAGTGAGTGACCATCCTGG	GCACCCCAAGACCTGCTTTAT
COL1	AGTCAGAGTGAGGACAGTGAATTG	CACATCACACCAGGAAGTGC
FN1	GGAAAGTGTCCCTATCTCTGATACC	AATGTTGGTGAATCGCAGGT
MMP8	ATGTTCTCCCTGAAGACGCT	AGACTGATACTGGTTGCTTGGT
Β-ACT	CCAGAGGCGTACAGGGATAG	GAGAAGATGACCCAGGACTCTC
GAPDH	ACGGGAAGCTTGTCATCAAT	GGAGGGATCTCGCATTTCTT

## Data Availability

MDPI Research Data Policies.
